# Downregulation of testosterone production through luteinizing hormone receptor regulation in male rats exposed to 17α-ethynylestradiol

**DOI:** 10.1038/s41598-020-58125-0

**Published:** 2020-01-31

**Authors:** Po-Han Lin, Tsung-Hsien Kuo, Chih-Chieh Chen, Cai-Yun Jian, Chien-Wei Chen, Kai-Lee Wang, Yuh-Chen Kuo, Heng-Yi Shen, Shih-Min Hsia, Paulus S. Wang, Fu-Kong Lieu, Shyi-Wu Wang

**Affiliations:** 1 Institute and Department of Physiology, School of Medicine, National Yang-Ming University, Taipei, 11221 Taiwan; 2 School of Nutrition and Health Sciences, College of Nutrition, Taipei Medical University, Taipei, 11031 Taiwan; 3 Institute of Clinical Medicine, School of Medicine, National Yang-Ming University, Taipei, 11221 Taiwan; 4 Department of Nutrition, China Medical University, Taichung, 40402 Taiwan; 5 College of Human Development and Health, National Taipei University of Nursing and Health Sciences, Taipei, 11219 Taiwan; 6 Department of Nursing, Ching Kuo Institute of Management and Health, Keelung, 20301 Taiwan; 7 Department of Urology, Yangming Branch of Taipei City Hospital, Taipei, 11146 Taiwan; 8 Department of Rehabilitation, Cheng Hsin General Hospital, Taipei, 11212 Taiwan; 9 Graduate Institute of Metabolism and Obesity Sciences, College of Nutrition, Taipei Medical University, Taipei, 11031 Taiwan; 10 Medical Center of Aging Research, China Medical University Hospital, Taichung, 40402 Taiwan; 11 Department of Biotechnology, College of Health Science, Asia University, Taichung, 41354 Taiwan; 12 Department of Medical Research, Taipei Veterans General Hospital, Taipei, 11217 Taiwan; 13 Department of Physical Medicine and Rehabilitation, National Defense Medical Center, Taipei, 11490 Taiwan; 14 Aesthetic Medical Center, Department of Dermatology, Chang Gung Memorial Hospital, Taoyuan, 33378 Taiwan; 15 Department of Physiology and Pharmacology, College of Medicine, Chang Gung University, Taoyuan, 33302 Taiwan

**Keywords:** Endocrine reproductive disorders, Endocrine reproductive disorders

## Abstract

The pharmaceutical 17α-ethynylestradiol (EE2) is considered as an endocrine-disrupting chemical that interferes with male reproduction and hormonal activation. In this study, we investigated the molecular mechanism underlying EE2-regulatory testosterone release *in vitro* and *in vivo*. The results show that EE2 treatment decreased testosterone release from rat Leydig cells. Treatment of rats with EE2 reduced plasma testosterone levels and decreased the sensitivity of human chorionic gonadotropin (hCG). EE2 reduced luteinizing hormone receptor (LHR) expression associated with decreased cAMP generation by downregulation of adenylyl cyclase activity and decreased intracellular calcium-mediated pathways. The expression levels of StAR and P450scc were decreased in Leydig cells by treatment of rats with EE2 for 7 days. The sperm motility in the vas deferens and epididymis was reduced, but the histopathological features of the testis and the total sperm number of the vas deferens were not affected. Moreover, the serum dihydrotestosterone (DHT) level was decreased by treatment with EE2. The prostate gland and seminal vesicle atrophied significantly, and their expression level of 5α-reductase type II was reduced after EE2 exposure. Taken together, these results demonstrate an underlying mechanism of EE2 to downregulate testosterone production in Leydig cells, explaining the damaging effects of EE2 on male reproduction.

## Introduction

The endocrine system precisely mediates sexual development and reproductive ability through circulating sexual hormones, which act as signaling molecules to render adequate regulation. Endocrine-disrupting chemicals (EDCs) are natural or industrial chemicals that are released into the environment through industries, livestock activities, domestic wastewater, and hospital effluence^1,2^. Numerous studies have indicated that EDCs interfere with endogenous endocrine processes by mimicking hormonal action, leading to infertility, metabolic syndrome, and the rise of cancer incidence^3–5^. In humans, increasing studies have indicated that EDCs have detrimental effects on the reproductive system, resulting in poor sperm quality and testicular dysgenic occurrence in men^6,7^, and disorders of ovulation and the uterus as well as a rise in breast cancer incidences in women^8,9^. Hence, the risks of EDCs are a global health issue^10^.

EE2 has been reported to exert a higher estrogenic activity than estradiol (E2), and is extremely stable against oxidation by metabolism or degradation in the human body due to the property of the ethynyl group in the C_17_ position^11–13^. This characteristic potentially allows 17α-ethynylestradiol (EE2) to pass into the environment^14,15^. Therefore, it is reasonable to consider environmental EE2 to be an EDC and have deleterious effects on the health of both animals and human beings^15^. The pharmaceutical EE2 is a synthetic estrogen widely used as an oral contraceptive pill, but abundant animal studies have also reported that long-term exposure to EE2 interferes with the biological function and the development of the reproductive system in female rats^16–18^ as well as affects the immune system^19^. Notably, a previous animal study reported that abnormal development of the external genitalia was observed in female offspring exposed to EE2 at 50 μg/kg during the neonatal period. The offspring of female rats exhibited an irregular estrus cycle, including persistent estrus, which reduces reproductive capacity in pre-middle age^20^. Furthermore, there are fewer studies investigating the effect of EE2 exposure on the male reproductive system. Although it has been reported that the androgen-dependent development of the reproductive tract in male rat offspring is interfered when the pregnant mother has been exposed to EE2^21^, there is still a lack of a comprehensive profile of the effects of EE2 on the male reproductive system, and its regulatory mechanisms are still unclear.

There are a large number of studies evaluating the effects of EE2, using a wide range of doses, on the endocrine and reproductive systems of aquatic organisms, causing complete feminization of male fish^22,23^, decreased sperm quality^24^ and reduced mating behavior^25^. A previous study has reported that EE2 could alter testosterone production in male fish by changing steroidogenic enzyme activities^26^. To assess whether EE2 can also affect the mechanism of testosterone production through alteration of steroidogenic enzyme activities in mammals, we designed, in the present study, an animal study to evaluate the effects of exposure to EE2 on the steroidogenic pathway activity in rat Leydig cells.

Our results showed that testosterone production was decreased by the treatment of Leydig cells with EE2. A consistent result was also observed by the treatment of rats with EE2 for 7 days. Its underlying molecular mechanism was a reduction of luteinizing hormone receptor (LHR)-regulatory activity, which in turn suppressed the process of steriodogenesis. In addition, the reproductive system of male rats was affected by EE2 exposure. These findings present a valuable insight into the mechanism underlying EE2-induced reduction of testosterone production.

## Results

### Inhibitory effects of EE2 on testosterone release in rat primary Leydig cells

**Figure 1:**
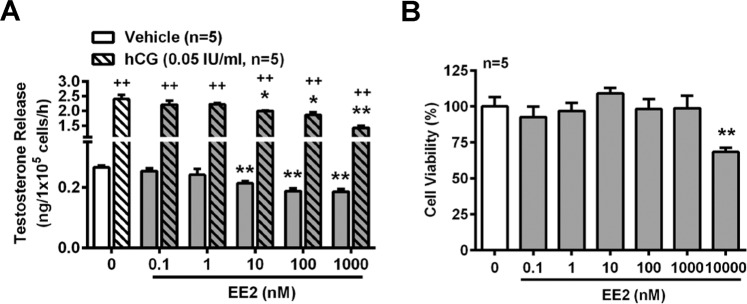
EE2 treatment reduces testosterone release in normal rat primary Leydig cells. EE2 inhibits testosterone release *in vitro*. (**A**) RIA for analyzing the concentration of testosterone in normal rat primary Leydig cells. Cells (1 × 10^5^ cells per tube) were treated with EE2 (0.1–1000 nM) in the absence or presence of hCG (0.05 IU/ml) for 1 h. At the end of the incubation, culture media were collected for testosterone RIA. (**B**) Cell survival assay. Rat primary Leydig cells (1 × 10^5^ cells per well) were seeded in a 96-well microplate and rested for 12 h. Subsequently, cells were treated with EE2 at different concentrations (0.1–10,000 nM) for 1 h. Then, the survival rate was measured by the MTT assay. Data represent means ± SEM (n = 5). *P < 0.05, **P < 0.01 as compared with the EE2-untreated group. ^**++**^*P* < 0.01 as compared with the untreated corresponding group.

To determine whether testosterone release from Leydig cells was suppressed by EE2 treatment, the primary rat Leydig cells were isolated from adult normal male rats and treated with EE2 using serial concentrations (0.1, 1.0, 10, 100, 1000 nM) for 1 h before measuring the testosterone concentration in the culture medium. The results showed that the basal level of testosterone released into the culture medium was decreased by exposure of the rat Leydig cells to EE2 at the concentrations from 10 to 1000 nM. Treatment with human chorionic gonadotropin (hCG), an analog of LH, was used to demonstrate that the cells were alive and had steroidogenic activity. We found that the level of testosterone release was increased by hCG treatment. However, this stimulated effect was reduced in the presence of EE2 at 10 to 1000 nM (Fig. 1A). In addition, to confirm that the results of EE2-inhibited testosterone release in the Leydig cells were not due to cell death caused by a high dosage of EE2, an MTT assay was conducted to examine the cell viability by treatment of the cells with EE2 at concentrations from 0.1 to 10,000 nM for 1 h. The results showed that the range of EE2 from 0.1 to 1000 nM did not cause any significant cell death as compared with the vehicle control. However, cell viability was reduced by treatment with 10,000 nM EE2 (Fig. 1B).

**Figure 2:**
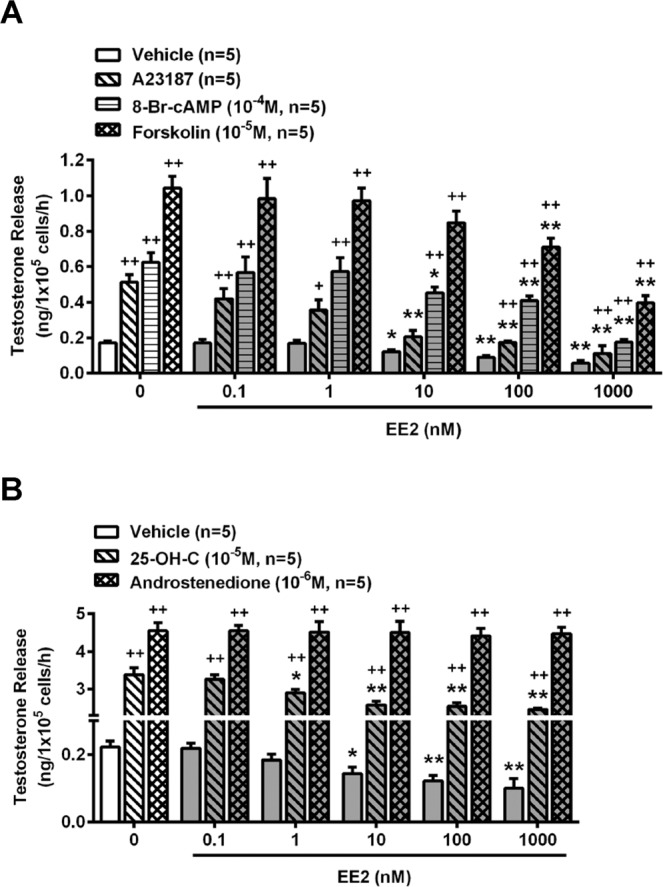
EE2 treatment suppresses adenylyl cyclase- and calcium-mediated testosterone release in normal rat primary Leydig cells. (**A**) Representative result of the effect of EE2 on testosterone release from normal rat primary Leydig cells (1 × 10^5^ cells per tube). Cells were treated with EE2 (0.1–1000 nM) in the presence of A23187 (10^−5^ M), 8-Br-cAMP (10^−4^ M) or forskolin (10^−5^ M) for 1 h. (**B**) Treatment with EE2 in the presence of 25-OH-C (10^−5^ M) or androstenedione (10^−6^ M) for 1 h was further evaluated for the molecular activity which is involved in the steroidogeneic pathway. At the end of the incubation, culture media were collected for testosterone RIA. Data represent means ± SEM (n = 5–6). *P < 0.05, **P < 0.01 as compared with the EE2-untreated group. ^+^*P* < 0.05, ^++^*P* < 0.01 as compared with the vehicle corresponding group. 25-OH-C, 25-hydroxycholesterol.

It has been reported that the activity of the cyclic AMP (cAMP) pathway and intracellular calcium level are increased in the mechanism of testosterone production^27^. Inasmuch as EE2 inhibited testosterone release from rat primary Leydig cells, we further examined the mechanism of EE2 action on the steroidogenesis of testosterone. Our results showed that treatment of the cells with 8-Br-cAMP (a permeable analog of cAMP), forskolin (an activator of adenylyl cyclase) or A23187 (a calcium ionophore) increased testosterone release, as compared with the vehicle, in the cells. However, these stimulatory effects on testosterone release were all reduced by co-treatment with EE2 at concentrations from 10 to 1000 nM (Fig. 2A).

To examine how EE2 was involved in interfering with testosterone biosynthesis, the two critical steps of steroidogenesis were detected. Treatment with 25-hydroxycholesterol (25-OH-C) was used to evaluate the capacity of cholesterol transportation in the steroidogenic pathway. The results showed that the level of testosterone was increased by 25-OH-C treatment, but this increased effect was suppressed in the presence of EE2 at 1 to 1000 nM (Fig. 2B). In addition, cells were administered with androstenedione for evaluating the activity of 17β- hydroxysteroid dehydrogenase (HSD). We found that the level of testosterone was not affected by co-treatment with EE2 and androstenedione (Fig. 2B).

### Role of LHR in EE2-induced decrease in testosterone release in male rats

**Figure 3:**
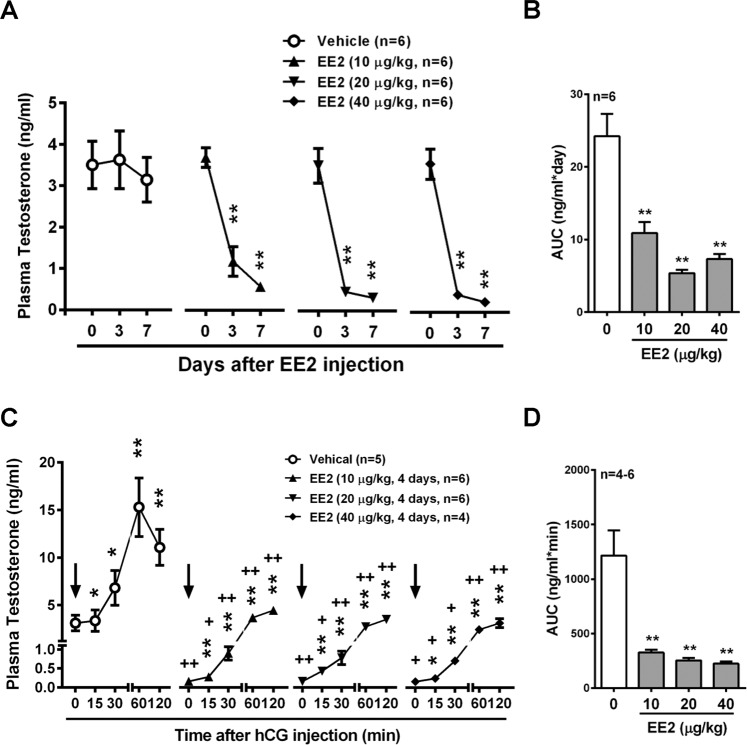
EE2 treatment reduces circulatory testosterone concentration through abolishing LH-mediated signaling. EE2 inhibits circulatory testosterone level *in vivo*. (**A**) Representative result of RIA of plasma testosterone concentration from male rats upon subcutaneous injection with EE2 once daily for 1 week. Blood samples were harvested on days 0, 3 and 7 by tail artery collection under anesthesia. Plasma samples were collected at the end of time points, and then the plasma was isolated for analysis of the concentration of testosterone using RIA (n = 6). (**B**) Quantification of the area under curve (AUC) during the period of EE2 injection is shown. (**C**) Representative result of RIA of plasma testosterone concentration on day 4 of EE2 injection. Rats were administered with hCG (5 IU/kg, arrow) through the right jugular vein (RJV). Blood was collected through the RJV at each time interval. Plasma samples were isolated and the concentration of testosterone was measured using RIA (n = 4-6). (**D**) Quantification of the AUC during hCG administration is shown. Data represent means ± SEM. *P < 0.05, **P < 0.01 as compared with the EE2-untreated group. ^**+**^*P* < 0.05, ^**++**^*P* < 0.01 as compared with the vehicle corresponding group.

To examine whether EE2 interfered with the circulatory concentration of testosterone, the male rats were treated with EE2 before detecting the plasma concentration of testosterone. Compared to the control group, a dramatic decrease in the testosterone level of approximately 70% was observed after injection for 3 days with EE2 (Fig. 3A). The area under curve (AUC) was measured as an index of EE2 effect on plasma testosterone production. The AUCs of all EE2-injected groups were significantly decreased as compared with the control group (Fig. 3B). We further examined the effects of EE2 on LH-mediated testosterone production *in vivo*. Rats were infused with hCG through the right jugular vein on day 4 post-EE2 injection. The plasma testosterone concentration was increased by hCG infusion in control rats, and reached a maximum level at 60 min after hCG administration (Fig. 3C). However, this stimulatory effect was attenuated in all EE2-injected groups. When hCG had been administered for 60 min in EE2 exposure groups, the increased testosterone levels were reduced to the basal level of the control group (Fig. 3C). These results were in accordance with the finding that the AUCs of all EE2-injected rat groups were significantly decreased as compared with the control group (Fig. 3D).

**Figure 4:**
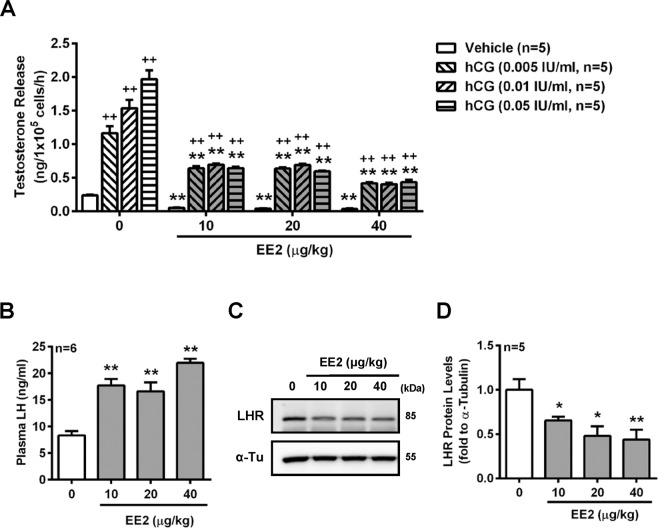
Detection of the role of LH in male rats injected with EE2. After EE2 injection for 7 days, rats were sacrificed under anesthesia. The Leydig cells were purified from the testes. (**A**) Representative result of RIA of the concentration of testosterone release. Cells (1 × 10^5^ cells per tube) were treated with the serial dosages of hCG (0.005, 0.01 or 0.05 IU/ml) for 1 h. At the end of the incubation, culture media were collected for testosterone RIA (n = 5). (**B**) Quantification of plasma LH level. Blood samples were collected through the abdominal aortic artery. Plasmas were isolated, the concentration of LH and measured by ELISA (n = 6). (**C**) Representative result and (**D**) quantification of the Western blot for LHR in Leydig cells. Leydig cells (3 × 10^6^ cells) were incubated in culture medium for 1 h, and then cells were harvested for performing immunoblot analysis of LHR, and normalized to α-tubulin (α-Tu) expression (n = 5). Data represent means ± SEM. *P < 0.05, **P < 0.01 as compared with the EE2-untreated group. ^**+**^*P* < 0.05, ^**++**^*P* < 0.01 as compared with the vehicle corresponding group.

The plasma level of testosterone was reduced in rats by the treatment with EE2 as described above. To understand the molecular mechanism of EE2 regulation on steroidogenesis in Leydig cells, we further isolated primary Leydig cells from rats exposed to EE2 for 7 days and then evaluated hCG effects on testosterone release. The results showed that treatment of the cells with hCG increased testosterone release in a dose-dependent manner in the control group, but this stimulatory effect was inhibited in all three EE2-injected groups (Fig. 4A). Following the negative feedback regulation, the opposite results showed that the plasma LH levels were increased by treatment of rats with EE2 for 7 days (Fig. 4B). Therefore, we further evaluated the effect of EE2 on the expression of LHR in rat Leydig cells. The results showed that the expression of LHR was downregulated in Leydig cells of rats injected with EE2 (Fig. 4C, D) as compared with the control group.

### Inhibition of LHR-induced adenylyl cyclase-cAMP regulation in Leydig cells of rats injected with EE2

**Figure 5:**
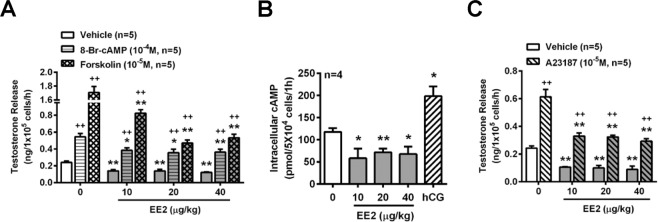
Adenylyl cyclase- and calcium-regulative signaling pathways are the major target of EE2 to inhibit testosterone production. After EE2 injection for 7 days, Leydig cells were isolated from the sacrificed EE2-injected rats. (**A**) RIA of the concentration of testosterone in the cells (1 × 10^5^ cells per tube) upon treatment with 8-Br-cAMP (10^−4^ M) or forskolin (10^−5^ M) for 1 h. At the end of the incubation, culture media were collected for testosterone RIA (n = 5). (**B**) Quantification of intracellular cAMP levels. Cells (5 × 10^4^ cells/tube) were pretreated with IBMX (0.5 mM) for 1 h, and then the medium was removed and replaced with a medium containing IBMX (0.5 mM) for incubation for an additional 1 h. Co-treatment with hCG (0.05 IU/ml) was used as a positive control in this study. At the end of incubation, the culture media were collected for detecting the intracellular cAMP by ELISA (n = 4). (**C**) The result is presented as the change in testosterone concentration in Leydig cells (1 × 10^5^ cells per tube) treated with A23187 (10^−5^ M) for 1 h. At the end of the incubation, culture media were collected for testosterone RIA (n = 5). Data represent means ± SEM. *P < 0.05, **P < 0.01 as compared with the EE2-untreated group. ^+^*P* < 0.05, ^++^*P* < 0.01 as compared with the vehicle corresponding group.

To further verify whether EE2 inhibited testosterone production through downregulation of the LHR regulatory pathway, the effects of the downstream pathway of LHR in Leydig cells were examined. The results showed that 8-Br-cAMP or forskolin-enhanced testosterone release was significantly decreased in all groups of Leydig cells isolated from rats injected with EE2 (Fig. 5A). We further monitored the adenylyl cyclase activity after determining the concentration of intracellular cAMP synthesis, which is the important secondary signaling transduction factor for the LHR-mediated steroidogenic pathway in the Leydig cells. Interestingly, the concentrations of intracellular cAMP were also decreased in Leydig cells of rats injected with EE2 as compared with the control group (Fig. 5B). The fact that the treatment of Leydig cells from control rats treated with hCG increased cAMP production was used as a positive control (Fig. 5B). In addition, the enhanced testosterone release from the Leydig cells following treatment with A23187 was also inhibited in EE2-injected groups (Fig. 5C).

### Inhibition of steroidogenesis in Leydig cells of rats injected with EE2

**Figure 6:**
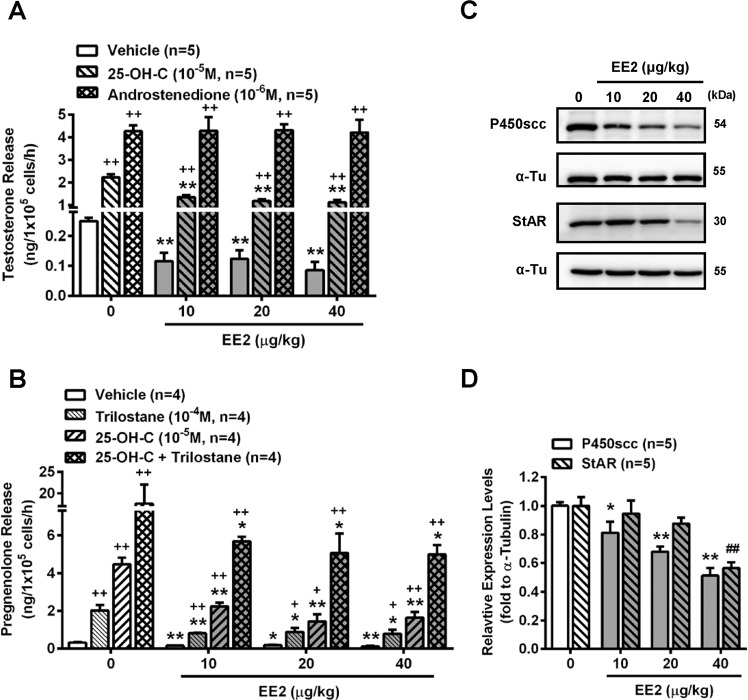
Injection of rats with EE2 reduced the enzyme activities of testosterone steroidogenesis in the Leydig cells. After treatment for 7 days, Leydig cells were isolated from the sacrificed EE2-injected rats. (**A**) Representative result of RIA of the concentration of testosterone in cells (1 × 10^5^ cells per tube) treated with 25-OH-C (10^−5^ M) or androstenedione (10^−6^ M) for 1 h. At the end of the incubation, culture media were collected for testosterone RIA (n = 5). (**B**) Determination of the effect of EE2 treatment on the activity of P450scc in Leydig cells through evaluating the level of pregnenolone. Cells were preincubated in the absence or presence of trilostane (10^−4^ M) for 1 h, and then treated with or without 25-OH-C (10^−5^ M) in the absence or presence of trilostane for an additional 1 h. At the end of the incubation, the pregnenolone concentration in the medium was measured (n = 4). (**C**) Western blot and (**D**) quantification of P450scc and StAR of Leydig cells (3 × 10^6^ cells) isolated from rats injected with EE2 for 7 days. Data represent means ± SEM. *P < 0.05, **P < 0.01 as compared with the EE2-untreated group. ^**+**^*P* < 0.05, ^**++**^*P* < 0.01 as compared with the vehicle corresponding group. 25-OH-C, 25-hydroxycholesterol; α-Tu, α-tubulin.

We further investigated whether the activity of steroidogenic enzymes was affected in Leydig cells isolated from rats injected with EE2. The results showed that 25-OH-C-increased testosterone release from the Leydig cells was attenuated in the presence of EE2, which suggested that a part of the steroidogenic pathway at the target of cholesterol was impaired (Fig. 6A). However, the androstenedione-induced increase in testosterone release was not altered by exposure to EE2, suggesting that the 17β-HSD activity might not be affected by EE2 exposure (Fig. 6A).

To confirm if the mechanism of cholesterol conversion during steroidogenesis of testosterone was impaired by EE2 treatment, the activity of cholesterol P450 side-chain cleavage enzyme (P450scc) was determined. In the presence of trilostane, an inhibitor of the 3β-HSD enzyme to accumulate pregnenolone, or 25-OH-C, the pregnenolone concentration increased in the Leydig cells, and was further increased by co-treatment with trilostane and 25-OH-C. However, these stimulations were all suppressed in Leydig cells isolated from EE2-injected rats, which suggested that the P450scc enzyme activity was inhibited by EE2 exposure (Fig. 6B). The P450scc protein level was also decreased in the Leydig cells of EE2-injected rats; moreover, the expression of steroidogenic acute regulatory protein (StAR), a transport protein which regulates movement of cholesterol into the inner membrane of the mitochondrion, was also downregulated (Fig. 6C, D).

### Effects of EE2 on the reproductive system of adult male rats

**Figure 7:**
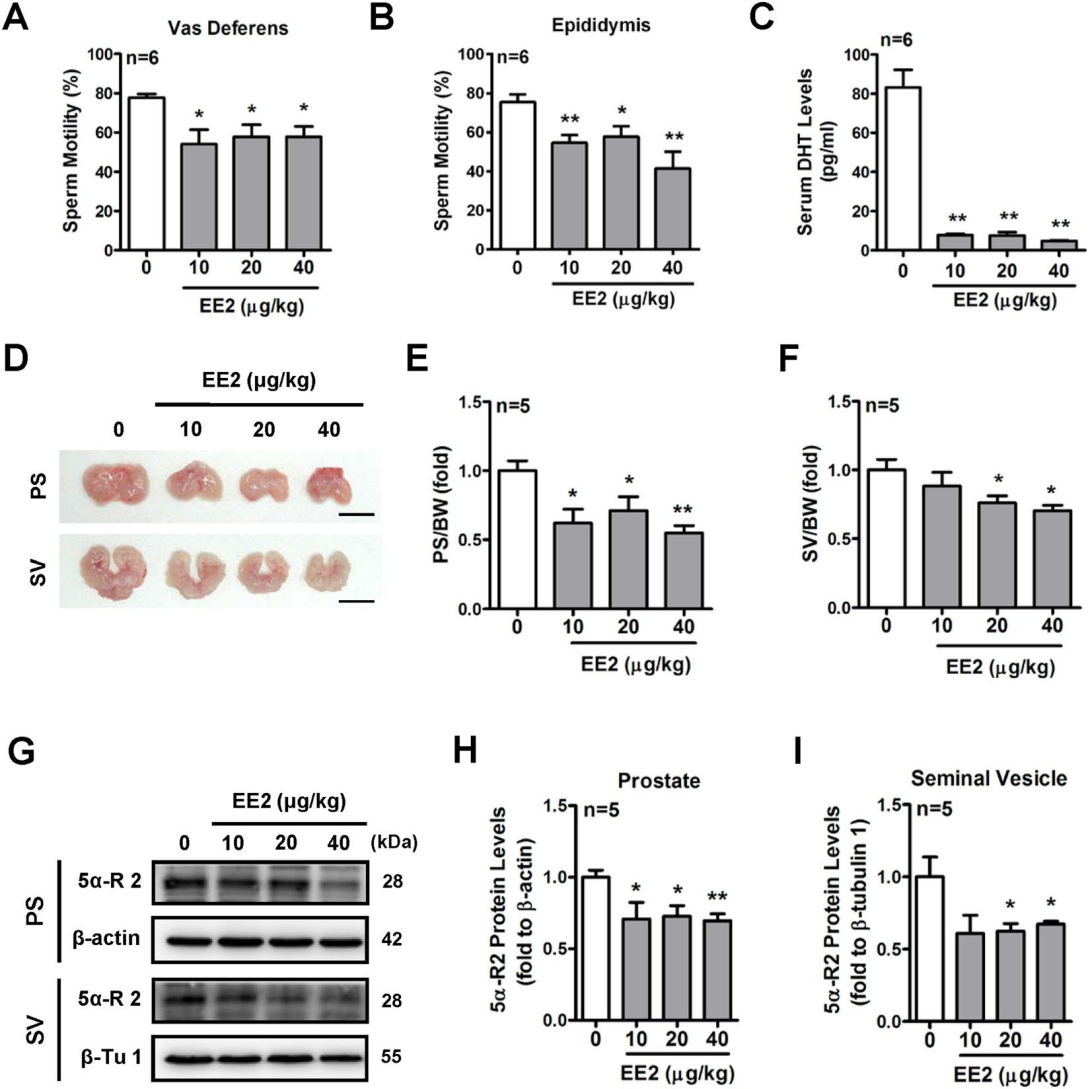
Inhibitory effects of EE2 on the reproductive system of male rats. After EE2 injection for 7 days, rats were sacrificed under anesthesia. Quantification of the sperm motility of the (**A**) vas deferens and (**B**) epididymis was measured under the microscope (n = 6). (**C**) Representative result of the concentration of serum DHT. Blood samples were collected, and serums were isolated. Serum DHT concentration was measured (n = 6). (**D**) Representative photos of the prostate glands and seminal vesicle from rats treated with EE2 for 7 days. Quantification of the weights of the (**E**) prostate glands and (**F**) seminal vesicle were normalized to the body weight (n = 5). (**G**) Western blot and (**H**,**I**) quantification of 5α-reductase type II of prostate glands and seminal vesicles isolated from rats injected with EE2 for 7 days. Prostate glands and seminal vesicles were lysed and the protein level of 5α-reductase type II was analyzed by immunoblotting and normalized to β-actin expression in the prostate glands and to β-tubulin 1 in the seminal vesicle. Data represent means ± SEM. *P < 0.05, **P < 0.01 as compared with the control group. PS, prostate gland; SV, seminal vesicle; 5α-R II, 5α-reductase type II; β-Tu 1, β-tubulin 1.

Male fertility and the maintenance of spermatogenesis require androgen mediation^28^. Based on the above results, the histopathological analysis of testes and the sperm quality in EE2-injected rats were further examined. The H&E staining results showed that the histological features of spermatogenesis and the total sperm number in rat vas deferens were not altered by treatment with EE2 for 7 days (Supplementary Fig. S1A and B). However, the sperm motility in both the vas deferens and epididymis was found to be decreased after EE2 exposure as compared with the control group (Fig. 7A, B). The circulatory DHT levels were parallel with the plasma testosterone levels in the EE2-injected rats (Fig. 7C). It has been reported that the testis, prostate, and seminal vesicle are the DHT target organs for supporting their biological functions^29^. Therefore, we further investigated whether these DHT target organs were affected by EE2 exposure. The results showed that the weights of the prostate and seminal vesicle decreased in EE2 rats (Fig. 7D to F), but there was no alteration in the weights of testes, in which the mean weights of testis were 3.30 ± 0.05 g, 3.11 ± 0.08 g, 3.16 ± 0.10 g, and 3.15 ± 0.09 g in the control group and in rats injected with EE2 at 10, 20, and 40 μg/ml, respectively. 5α-reductase has been known to convert testosterone to DHT in the prostate gland and seminal vesicles^30,31^. We next examined whether the protein levels of type II 5α-Reductase (5α-R II), the major isoform, in both the prostate gland and seminal vesicle were associated with the reduction of plasma DHT concentrations in EE2-injected rats. The results showed that the expression of type II 5α-reductase protein in both the prostate gland and the seminal vesicle was decreased by EE2 exposure (Fig. 7G to I).

## Discussion

EE2 is a potent estrogenic compound with a higher binding affinity for estrogen receptors than natural E2. EE2 is one of the most commonly used medicines for female contraception, but approximately 40% of the EE2 dose is excreted in the urine and feces^32^. Because EE2 possesses estrogenic properties, it has been considered as an EDC when it is excreted into the environment through wastewater^33^. Previous studies indicated that long-term exposure to EDCs is accompanied by a number of diseases and dysfunctions to become a global challenge in health management, causing great financial burden in the United States^34^ and Europe^35^. Epidemiological studies have reported that the reduction of semen quality in men of reproductive age is associated with the environments^36–38^. Particularly, EDCs exposure have been identified to have an impact on the male reproductive system^39^.

It has been reported that exposure to EE2 could impair the process of development and the reproductive system in aquatic organisms^15,40,41^. Recently, the effect of EE2 on the male reproductive system in mammals has also been reported^21^, but the specific inhibitory mechanism of EE2 is still unclear. In the present study, we demonstrated that EE2, in the 0.1–1000 nM concentration range, decreased testosterone release from the primary culture of rat Leydig cells. The effect of EE2 *in vivo* was verified and the results showed that the plasma testosterone level was significantly decreased in rats injected with EE2 for 7 days. Moreover, the hCG-evoked increase in plasma testosterone level was retarded in male rats exposed to EE2. Importantly, we found that the inhibitory mechanism of EE2 was through decreasing LHR expression, which in turn reduced adenylyl cyclase activity and the generation of downstream cAMP. The intracellular calcium regulatory signaling was also lowered in Leydig cells of rats injected with EE2. Furthermore, the mechanism of transportation of cholesterol into mitochondria was reduced because of decreased protein levels of StAR in the Leydig cells of EE2-injected rats. Subsequently, both the protein level and enzyme activity of P450scc were also decreased by EE2 exposure. Furthermore, decreased sperm motility and prostate and seminal vesicle atrophy were also demonstrated in the EE2-injected rats, indicating EE2-induced damage to the reproductive system.

Gonadal steroid hormones are released and modulated by the hypothalamus-pituitary-gonadal (HPG) axis, which connects the central nervous system with the hormone system^42^. Testosterone is a major testicular steroid produced by Leydig cells, and is released in response to LH secreted from the anterior pituitary gland^43,44^. Testosterone can be converted to estradiol by aromatase or converted to DHT by 5α-reductase in the local gonadal organs^45^. It is also reported that higher plasma estradiol levels inhibit testosterone production through a negative feedback regulation on the LH-stimulating pathway^46,47^. In the present study, we found that the hCG-evoked increase in plasma testosterone was significantly suppressed in male rats injected with EE2, suggesting that downregulation of the LHR regulatory pathway might be involved in the reduced testosterone production caused by EE2. Furthermore, LH stimulates testosterone production through activating adenylyl cyclase to promote its downstream cAMP generation^48^ and also by intracellular calcium level^27,49^. Both are involved in regulating steroidogenesis-associated genes activation. Our results also showed that the expression of LHR and its downstream cAMP generation were downregulated in the Leydig cells of EE2-injected rats. Moreover, EE2-exposed rats exhibited reduced responses to forskolin-, 8-Br-cAMP- or A23187-induced increase in testosterone production in the Leydig cells. These results suggest that EE2 inhibited the Leydig cells to release testosterone through interfering with the LHR-adenylyl cyclase-cAMP and intracellular calcium-regulatory pathways.

The steroidogenic mechanism of testosterone involves multiple steps, which are catalyzed by several enzymes. In the present study, we further evaluated whether EE2 interfered with the mechanism of testosterone biosynthesis. Our results showed that EE2 did not affect the androstenedione-induced increase in testosterone release, suggesting that the ability of 17β-HSD might not be affected in the Leydig cells of EE2-injected rats. However, EE2 treatment reduced the 25-OH-C-induced testosterone release, suggesting that the enzyme, which is involved in the transfer of cholesterol into mitochondria, might be impaired when exposed to EE2. Therefore, we further examined whether the P450scc enzyme activity in Leydig cells was impaired after exposure to EE2. The results showed that the P450scc protein levels and enzyme activities were significantly decreased in the Leydig cells of rats injected with EE2. Meanwhile, we found that the StAR protein levels were also suppressed, suggesting that the ability of the StAR protein to move the cholesterol into mitochondria was impaired after EE2 exposure. In agreement with our findings, Garcia-Reyero and colleagues reported that male fathead minnows, when exposed to EE2, showed downregulated StAR and P450scc mRNA expression^50^. Hogan and colleagues also reported that the testosterone level was diminished by depressing P450scc enzyme activity in EE2-exposed male estuarine killifish^26^. Taken together, these results suggest that EE2 reduced testosterone release from the Leydig cells by downregulating steroidogenic enzymes in vertebrates.

The frequency of courtship-specific behavior in male zebrafish was hampered by exposure to EE2^51^. Moreover, EE2-exposed male pipefish presented lower mating behavior related to effects on their attractiveness to females^25^. Besides mating behavior, the effects of EE2 on the reproductive system have also been investigated in aquatic organisms. A previous study demonstrated that male fighting fish exposed to EE2 exhibited smaller gonads and fewer moles of intracellular ATP, resulting in a reduction in sperm motility^24^. Furthermore, it was reported that the male rainbow trout had an increased level of aneuploid sperm formation due to prolonged EE2 exposure for 50 days^52^. In the present study, the effect of EE2 on the reproductive system in male rats was investigated. We found that the histological features of spermatogenesis in the testis and the total sperm number in the vas deferens were not altered, but the sperm motility of the vas deferens and epididymis was reduced by treatment of rats with EE2 for 7 days. In contrast to our finding, Iwase and colleagues reported that male rats orally administered with 0.1 or 0.3 mg/kg EE2 for 4 weeks exhibited lower sperm counts due to degenerative changes in spermatogenesis, but sperm motility was not altered^53^. Significantly, reproductive ability was lost in male rats exposed to EE2 at the higher dosages of 3 or 10 mg/kg^53^. It is noteworthy that in male rats exposed to EE2, prostate and seminal vesicle atrophy was induced in our results, which is similar to the findings of Iwase and colleagues^53^. The above evidence collectively suggests that exposure to EE2 causes damage to the male reproductive system, resulting in reduced fertility.

**Figure 8:**
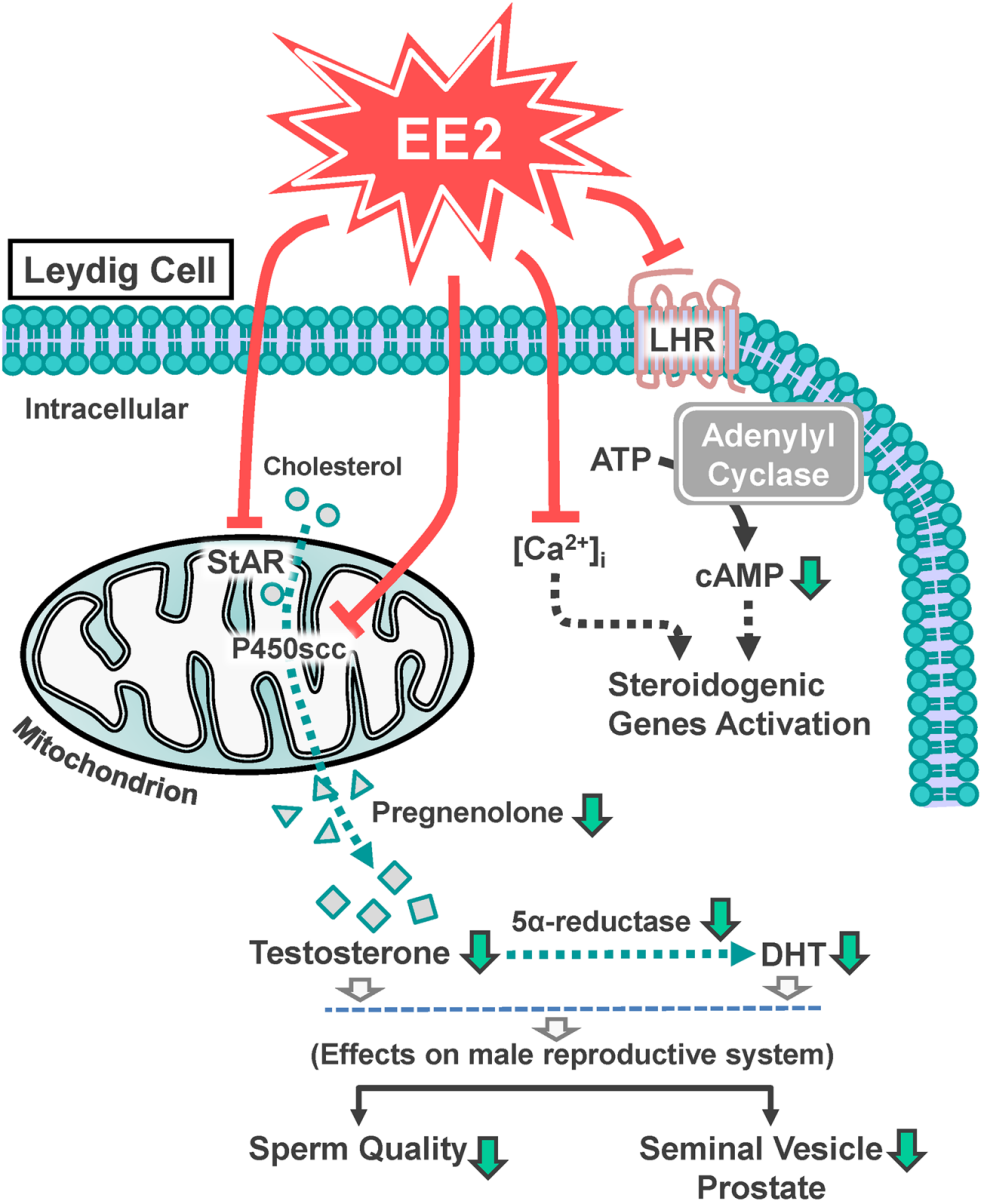
Schematic representation of the cellular model for the inhibitory effect of EE2 on the male reproductive system through downregulation of testosterone production in rat Leydig cells. EE2 inhibited LHR protein expression, and subsequently downregulated cAMP activity, which in turn decreased steroidogenic genes expression. EE2 also inhibited StAR protein expression and P450scc protein level and activity, which in turn suppressed the production of pregnenolone, testosterone and DHT, leading to a hampered male reproductive system, including reduced size of the prostate and seminal vesicle and reduced sperm quality.

In conclusion, the results of the present study show that EE2 exposure attenuated testosterone production through downregulation of LH receptor-mediated and calcium-activated steroidogenic pathways. Moreover, EE2 inhibited the initiative step of steroidogenesis via suppressing the expression of StAR protein and the activity of P450scc, subsequently reducing the production of pregnenolone, and finally reduced testosterone and DHT production. We also found that EE2 hampered the male reproductive system, including reduction in the size of the prostate gland and seminal vesicle and declined in sperm quality. Based on the results of the present study, we propose a schematic model of the mechanism underlying EE2-inhibited testosterone production in rat Leydig cells, as shown in Fig. 8.

## Materials and Methods

### Animals

Male Sprague-Dawley rats age two to three months old were purchased from the Laboratory Animal Center of National Yang-Ming University (Taipei, Taiwan) and BioLASCO (Taipei, Taiwan). The animals were housed in a room with 14 hours of artificial illumination (06:00–20:00) and controlled temperature (22 ± 2 °C). Food and water were given *ad libitum*. All animal experimental protocols were according to the guide for the care and use of laboratory animals (8th edition) and the Institutional Animal Care and Use Committee (IACUC). National Yang-Ming University (Permit Number: 1040409) and Taipei Medical University (Permit Number: LAC-2019-0078) approved all animal experiments conducted.

#### Experiment design I - EE2 administration *in vivo*

The dosage of EE2 from 0.02 to 200 μg/kg was selected for examining its biological activity in the animal study. However, significant effects of EE2 treatment have been observed among concentrations from 2 to 50 μg/kg^16,19,21,54^. Therefore, in this study, EE2 (Sigma-Aldrich, St Louis, MO, USA) was dissolved in sesame oil (Sigma-Aldrich). Male rats were randomly divided into four groups. Rats were subcutaneously injected with sesame oil as the control group. The other three groups represent the three individual concentrations of EE2 10, 20, or 40 μg/kg subcutaneously injected once daily for a week. Blood samples (1 ml) were harvested on days 0 (before EE2 injection), 3 and 7 by tail artery collection under anesthesia. Plasma and serum were separated from rat blood samples after centrifugation at 10,000 × *g* for 5 min, and then stored at −80 °C for further analysis.

#### Experiment design II – hCG administration *in vivo*

On day 4 after EE2 injection, rats were administered with hCG (5 IU/kg; Sigma-Aldrich) through the right jugular vein. Then blood samples were collected at 15, 30, 60, and 120 min after injection. The volume of blood samples (0.5 ml each) was collected at each time point. Plasma and serum samples were separated from blood after centrifugation at 10,000 × *g* for 5 min, and then stored at −80 °C for further analysis.

All experiment rats were sacrificed under anesthesia using sodium pentobarbital (40 mg/kg; Koch-Light. Lab. Ltd., Colnbrook, Bucks, England). Blood samples were collected through abdominal aortic artery exsanguinations. The Leydig cells were purified from testes immediately. Prostate and seminal vesicle tissues were quickly excised and stored at −80 °C for further analysis.

### Catheterization of the right jugular vein (RJV)

Rats were catheterized using a polyethylene tubing (PE-50) ending in a segment of silastic tubing via the right jugular vein, under anesthesia with sodium pentobarbital (40 mg/kg; Koch-Light. Lab. Ltd.), as described previously^55,56^. The catheter was passed subcutaneously towards the nape, where it was allowed to protrude through a small incision. After surgery, the rats were allowed to recover for 1 day. Before experiments, the catheter was filled with heparin saline (100 IU/ml), without any air bubbles, and blood.

### Preparation of rat primary Leydig cells

The process of purification of rat primary Leydig cells was as described previously^57–59^. The testes were collected and decapsulated after the rats were sacrificed. The testicular interstitial cells were isolated by incubation in 1% BSA-HBSS containing collagenase (Sigma-Aldrich) at 34 °C for 15 min. The mixture was filtered, and hypotonic shock was performed twice. The supernatant was loaded onto the upper layer of a Percoll gradient (GE Healthcare Life Sciences, Pittsburgh, PA, USA) and was centrifuged at 4 °C, 1000 × *g* for 30 min. Leydig cells were located in the first 3–7 ml from the bottom. The layer of Leydig cells was collected and washed using a culture medium (1% BSA in Medium 199 with 25 mM HEPES, 2.2 g/ml NaHCO_3_, 100 IU/ml penicillin-G, 50 μg/ml streptomycin sulfate, 2550 IU/l heparin, pH 7.4). After repeated washing, the cell pellet was suspended in the same culture medium. Cell concentration and viability (over 95%) were determined using a trypan-blue counterstained method. For rest and stabilization, the Leydig cells (1 × 10^5^ cells/tube) were pre-incubated with the culture medium at 34 °C for 1 h. After the end of pre-incubation, cell media were removed by centrifugation and then the cell pellets were re-suspended in the challenged media as described in the figure legends. Finally, the media were collected for further analysis.

### Cell survival assay

The survival rate of rat Leydig cells after EE2 treatment was detected by MTT (3-[4,5-dimethyl-2-thiazolyl]-2,5-diphenyl-2H-tetrazolium bromide, Sigma-Aldrich) assay. Cells (1 × 10^5^ cells/well) were seeded in a 96-well microplate with 100 μl culture medium. After cell attachment and resting for 12 h, the medium was removed and 100 μl challenged media containing serial doses of EE2 were added, before incubation for 1 h. Subsequently, the medium was removed again and replaced by serum-free culture medium with 1 mg/ml MTT. The cells were incubated for another 4 h. The medium was removed and crystal formazan dissolved in 50 μl DMSO (Sigma-Aldrich) was added. The optical density was measured by a microplate reader (TECAN Sunrise ELISA Reader, Männedorf, Switzerland) at 570 nm and 630 nm as the reference wavelength.

### Radioimmunoassay (RIA) of testosterone

The concentrations of testosterone in the culture medium and plasma were determined by RIA as previously described^60^. Using an anti-testosterone serum (No. W8) prepared by our laboratory, the sensitivity of the testosterone RIA was 2 pg per assay tube. The intra- and inter-assay coefficients of variation were 4.1% (n = 6) and 4.7% (n = 10), respectively.

### Enzyme-linked immunosorbent assay (ELISA) of intracellular cAMP and pregnenolone

The concentration of intracellular cAMP was measured by the cAMP ELISA kit (Cayman Chemical, Ann Arbor, MI, USA). After male rats injected with EE2 for 7 days, the Leydig cells were isolated from the testes. After pretreating the Leydig cells (1 × 10^5^ cells/tube) with 3-isobutyl-1-methylxanthine (IBMX; 0.5 mM, Sigma-Aldrich), an inhibitor of cAMP phosphodiesterase, for 1 h, cells were treated with IBMX in the absence or presence of hCG (0.05 IU/ml) for an additional 1 h. After centrifugation, the cell pellet had 150 μl HCl (0.1 M) added to it and was incubated at room temperature for 20 min. The mixture was centrifuged, and the supernatant was decanted into a new test tube for further analysis. Subsequent procedures were performed according to the manufacturer’s protocol. The absorbance values were measured by a microplate reader (TECAN) at 420 nm in 120 min.

The concentration of pregnenolone in the culture medium was measured as previously described^58^. A 96-well microplate was coated with pregnenolone-BSA (0.2 μg/200 μl/well in coating buffer). After coating and blocking, pregnenolone standards (Sigma-Aldrich) or samples (50 μl) in combination with the primary antibody (50 μl/well, 1:12,800 dilution) were added into the wells of the plate and incubated at 37 °C for 1 h. After discarding the contents of the wells, the plate was washed and incubated with a conjugated secondary antibody (200 μl/well, IgG-HRP, 1:5000 dilution) at 37 °C for 1 h. After washing, each well then had 3,3′,5,5′-tetramethylbenzidine substrate (TMB, 200 μl/well; Sigma-Aldrich) added to it under light-protected conditions at room temperature for 10 min. Finally, HCl (2 N) was applied to stop the reaction. The absorbance values were measured by a microplate reader (TECAN) at 450 nm and using 650 nm as the reference wavelength.

### Measurements of LH and DHT

The concentration of plasma LH was measured by the LH ELISA kit (Enzo Life Sciences, Farmingdale, NY, USA). In addition, the concentration of serum DHT was measured by the DHT ELISA kit (Immuno-Biological Laboratories Inc., Minneapolis, MN, USA). All procedures were performed according to the manufacturer’s protocol. The absorbance values were measured by a microplate reader (TECAN) at 450 nm.

### Protein preparation and Western blot analysis

Total cell and tissue protein levels were analyzed by Western blot, as described previously^60^. Briefly, quantitative amounts of samples (60 μg) were mixed with a sample buffer (0.125 M Tris-Cl pH 6.8, 20% glycerol, 4% sodium dodecyl sulfate (SDS), 10% 2-mercaptoethanol, 0.1% bromophenol blue) at a ratio of 1:1. After being boiled for 5 min, the mixture samples were separated by using 9.5% or 12% SDS-polyacrylamide gel electrophoresis and then transferred onto polyvinyl difluoride membranes (Millipore, Billerica, MA, USA). The membranes were blocked with 5% fat-free milk (Anchor, Auckland, NZ) in TBS-T (0.137 M NaCl, 20 mM Tris, 0.1% Tween-20, pH 7.6) for at least 1 h at room temperature. The membranes were incubated with a specific antibody against LHR (1:500; Santa Cruz Biotechnology, Santa Cruz, CA, USA), P450scc (1:10,000; Bioss Inc., Woburn, MI, USA), StAR (1:8000 dilution), α-tubulin (1:5000; Sigma-Aldrich), β-actin (1:5000; Sigma-Aldrich), and β-tubulin 1 (1:5000; Sigma-Aldrich) overnight at 4 °C. Subsequently, the membranes were incubated with appropriate secondary antibodies: horseradish peroxidase-conjugated goat anti-rabbit and/or goat anti-mouse IgG (1:10,000, Jackson ImmunoResearch Laboratories, West Grove, PA, USA) for 1 h. To quantify the intensity of the protein expression levels, the membranes were developed with enhanced chemiluminescence (PerkinElmer Life Sciences, Boston, MA, USA), and the visual signal was recorded by Luminescence Imaging System LAS-4000 (*GE* Healthcare Life Sciences). The band densities were determined as arbitrary absorption units using the Image-J software program.

### Sperm count and motility assay

The process of the sperm motility assay was modified from previous studies^61,62^. The rat cauda epididymis was punctured by scissors and 2 μl semen was mixed with 2 ml Medium 199 culture medium (containing 1% BSA and 1 g/L glucose). The rat vas deferens (~3 cm) was cut into four sections and placed in 3 ml Medium 199 culture medium (coating 1% BSA). After gently shaking for 10 min, the sperm was dissociated into the medium. The sperm suspension was mixed with 0.4% Trypan blue solution. The numbers of active and quiescent sperms were counted using a hemocytometer under a microscope.

### Haematoxylin and eosin (H&E) staining

At the end of the experiment, testes were fixed in 10% formalin and embedded in paraffin. Three-micrometer cross-sections were collected onto slides. Tissue sections were stained with haematoxylin and eosin (H&E) by Bio-Check Laboratories Ltd (Taipei, Taiwan). Images were captured at 200x magnification using the EVOS microscope (Thermo Fisher Scientific, Waltham, MA, USA).

### Statistical analysis

The quantitative values were represented as mean ± standard error of the mean (SEM). The difference among all groups was evaluated by one-way analysis of variance (ANOVA) followed by Student’s unpaired *t*-test, which was used for comparison between two groups, and Student’s paired *t*-test, which was used for comparison in the same group between pre- and post-treatments. Statistical significance was assigned at *P* < 0.05^63^. Statistical analysis was performed by using Prism version 6.0 software (GraphPad, San Diego, CA, USA).

## Supplementary information

Supplementary Information.
