# Supplementary material for: Downregulation of testosterone production through luteinizing hormone receptor regulation in male rats exposed to 17α-ethynylestradiol
Source: Sci Rep. 2020 Jan 31;10:1576. doi: 10.1038/s41598-020-58125-0 (PMC6994641; doi:10.1038/s41598-020-58125-0)
Supplement: Supplementary file 1 — Supplementary Information. [file 41598_2020_58125_MOESM1_ESM.pdf]

Supplementary Information:

Downregulation of testosterone production through luteinizing hormone receptor regulation in male rats exposed to 17α-ethynylestradiol

Po-Han Lin, Tsung-Hsien Kuo, Chih-Chieh Chen, Cai-Yun Jian, Chien-Wei Chen, Kai-Lee Wang, Yuh-Chen Kuo, Heng-Yi Shen, Shih-Min Hsia, Paulus S. Wang*, Fu-Kong Lieu*, Shyi-Wu Wang*

***Corresponding author:**

[1] Shyi-Wu Wang, Ph.D.

Department of Physiology and Pharmacology, College of Medicine,

Chang-Gung University, Taoyuan 33302, Taiwan.

Tel: +886-3-211-8800 ext.5253

Fax: +886-2-211-8700

E-mail: swwang@mail.cgu.edu.tw

[2] Paulus S Wang, Ph.D.

Medical Center of Aging Research, China Medical University Hospital,

Taichung 40402, Taiwan

Tel: +886-4-2205-2121 ext.7703

Fax: +886-2-2873-5558

E-mail: pswang3879@gmail.com

[3] Fu-Kong Lieu, M.D.

Department of Rehabilitation, Cheng Hsin General Hospital, Taipei 11212, Taiwan.

Tel: +886-2-2826-4400 ext.3806

Fax: +886-2-2826-4400 ext.3814

E-mail: ch2276@chgh.org.tw

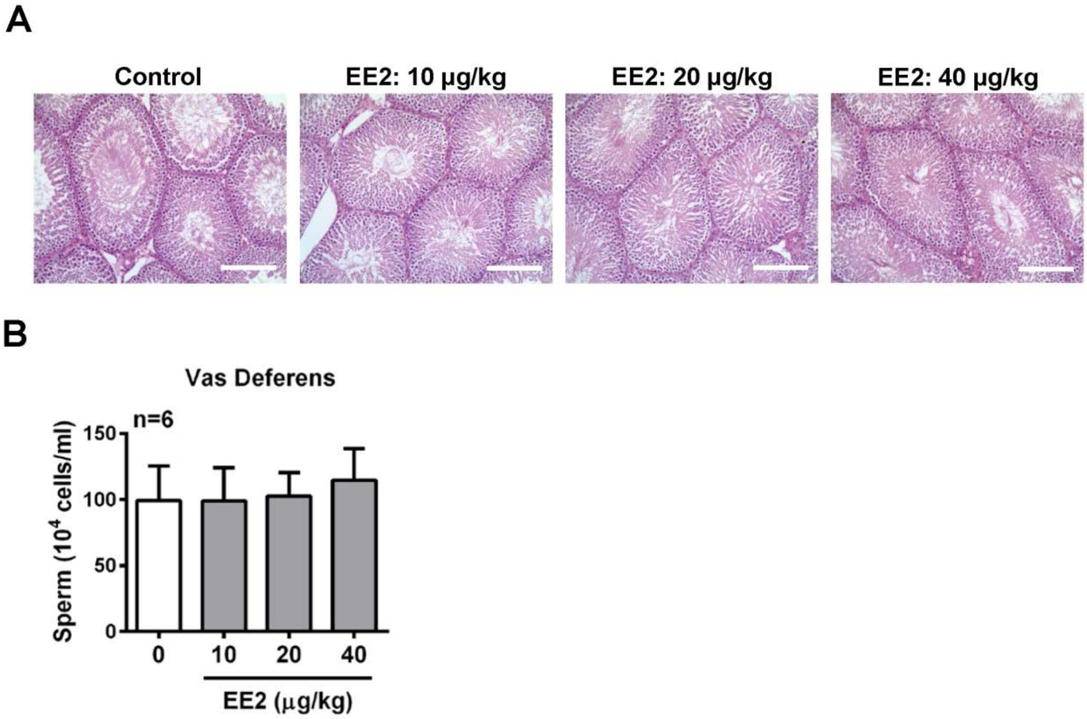

Supplementary Fig. S1. Injection of rats with EE2 was no alteration in the spermatogenesis and the total sperm number in the vas deferens. After EE2 injection for 7 days, rats were sacrificed under anesthesia. (A) Representative results of the haematoxylin and eosin (H&E) staining of the seminiferous tubule. Testis tissues were harvested and then formalin-fixed and paraffin-embedded. Three-micrometer cross-sections of the tissues were collected onto slides. The H&E staining was performed to observe the histopathological features. (magnification 200x; scale bar = 200 μm). (B) Quantification of the total sperm number in vas deferens. The number of sperm was countered under the microscope. One-Way ANOVA: *P* = 0.9639 (n = 6).

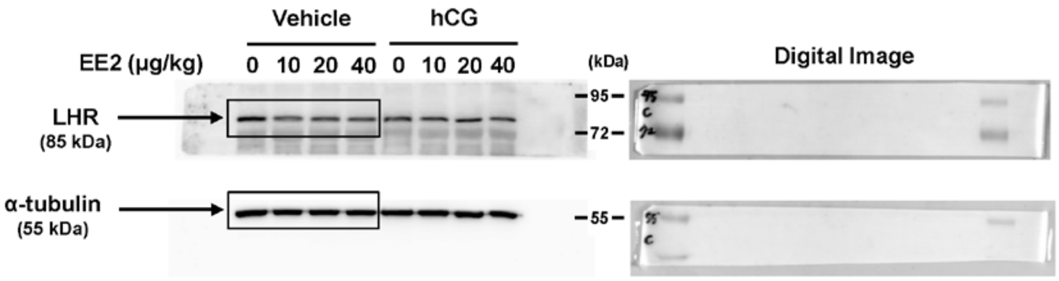

Supplementary Fig. S2. The original blot pictures of Fig. 4. in the text. Corresponding to the Fig. 4C. Size distribution of the molecular weight marker was shown in the digital images. The cropping lines and the molecular weight of proteins were indicated.

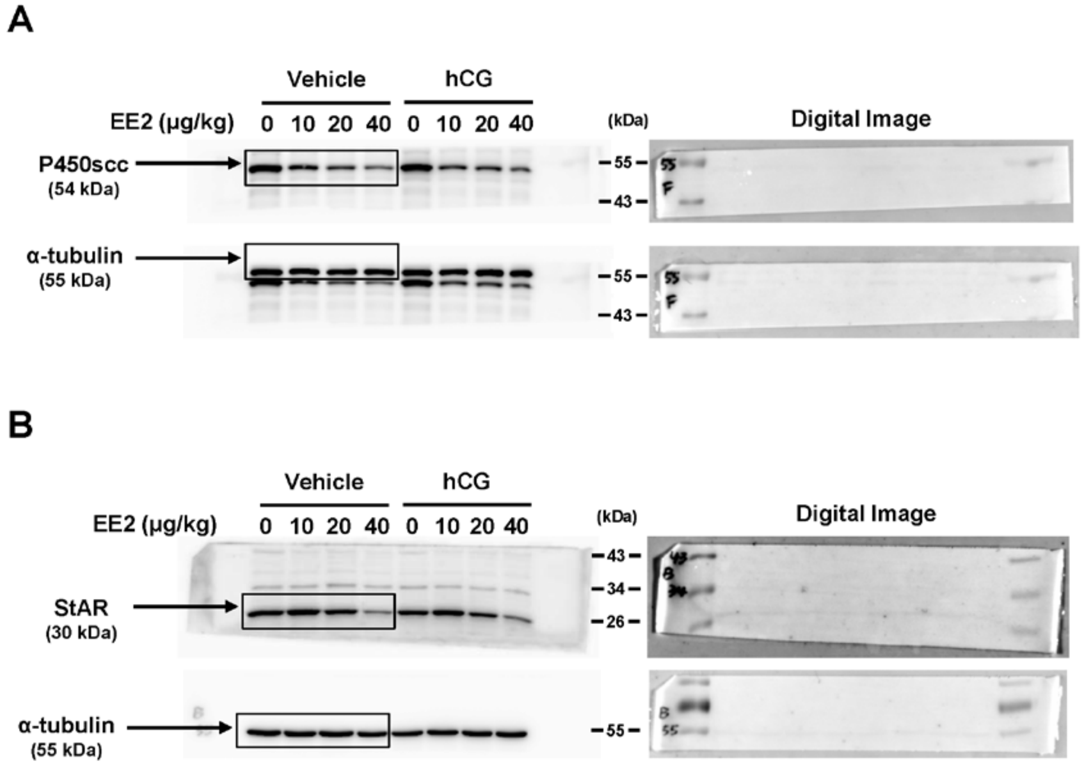

Supplementary Fig. S3. The original blot pictures of Fig. 6. in the text. (A) Corresponding to the section of P450scc in Fig. 6C. (B) Corresponding to the section of StAR in Fig. 6C. Size distribution of the molecular weight marker was shown in the digital images. The cropping lines and the molecular weight of proteins were indicated.

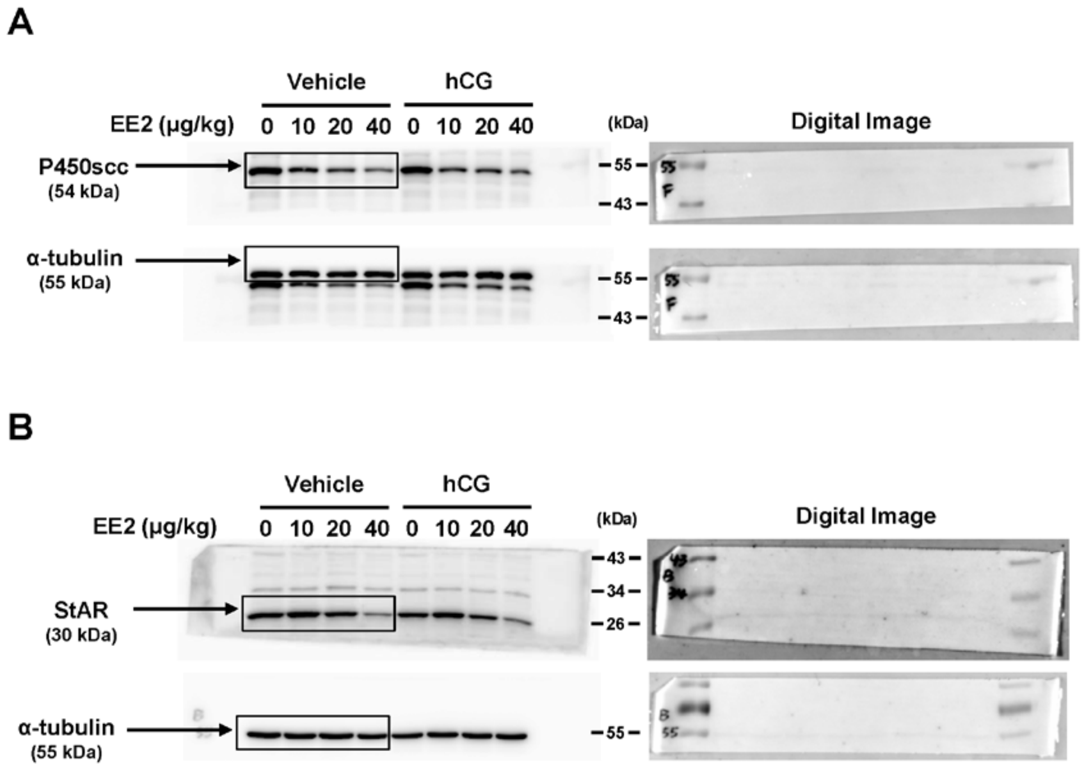

Supplementary Fig. S4. The original blot pictures of Fig. 7. in the text. Two individual rats of each group were shown in the membrane in the prostate gland and seminal vesicle, respectively. (A) Corresponding to the section of prostate gland in Fig. 7G. (B) Corresponding to the section of seminal vesicle in Fig. 7G. Size distribution of the molecular weight marker was shown in the digital images. The cropping lines and the molecular weight of proteins were indicated.
